# Keemei: cloud-based validation of tabular bioinformatics file formats in Google Sheets

**DOI:** 10.1186/s13742-016-0133-6

**Published:** 2016-06-13

**Authors:** Jai Ram Rideout, John H. Chase, Evan Bolyen, Gail Ackermann, Antonio González, Rob Knight, J. Gregory Caporaso

**Affiliations:** Center for Microbial Genetics and Genomics, Northern Arizona University, Flagstaff, AZ 86011 USA; Department of Pediatrics, University of California San Diego, San Diego, CA 92093 USA; Department of Computer Science and Engineering, University of California San Diego, San Diego, CA 92093 USA; Department of Biological Sciences, Northern Arizona University, Flagstaff, AZ 86011 USA

**Keywords:** Data validation, Tabular file format, Spreadsheet, QIIME, Metadata, Cloud, Plugin

## Abstract

**Background:**

Bioinformatics software often requires human-generated tabular text files as input and has specific requirements for how those data are formatted. Users frequently manage these data in spreadsheet programs, which is convenient for researchers who are compiling the requisite information because the spreadsheet programs can easily be used on different platforms including laptops and tablets, and because they provide a familiar interface. It is increasingly common for many different researchers to be involved in compiling these data, including study coordinators, clinicians, lab technicians and bioinformaticians. As a result, many research groups are shifting toward using cloud-based spreadsheet programs, such as Google Sheets, which support the concurrent editing of a single spreadsheet by different users working on different platforms. Most of the researchers who enter data are not familiar with the formatting requirements of the bioinformatics programs that will be used, so validating and correcting file formats is often a bottleneck prior to beginning bioinformatics analysis.

**Main text:**

We present Keemei, a Google Sheets Add-on, for validating tabular files used in bioinformatics analyses. Keemei is available free of charge from Google’s Chrome Web Store. Keemei can be installed and run on any web browser supported by Google Sheets. Keemei currently supports the validation of two widely used tabular bioinformatics formats, the Quantitative Insights into Microbial Ecology (QIIME) sample metadata mapping file format and the Spatially Referenced Genetic Data (SRGD) format, but is designed to easily support the addition of others.

**Conclusions:**

Keemei will save researchers time and frustration by providing a convenient interface for tabular bioinformatics file format validation. By allowing everyone involved with data entry for a project to easily validate their data, it will reduce the validation and formatting bottlenecks that are commonly encountered when human-generated data files are first used with a bioinformatics system. Simplifying the validation of essential tabular data files, such as sample metadata, will reduce common errors and thereby improve the quality and reliability of research outcomes.

**Electronic supplementary material:**

The online version of this article (doi:10.1186/s13742-016-0133-6) contains supplementary material, which is available to authorized users.

## Findings

### Background

Many bioinformatics applications require human-generated tabular text files as input and have specific requirements for how those data are formatted. A common example is metadata describing a collection of biological samples (i.e. a *sample metadata mapping file*), such as ISA-TAB-based file formats [1]. For example, in a human microbiome survey, this file would map unique sample identifiers [2] to descriptions of each sample for minimum information standards compliance [3], study-specific parameters such as host identifier and disease state and technical information such as, for marker genes, the polymerase chain reaction primer pair that was used to amplify and sequence the gene reading out the community profile, and, for shotgun metagenomics, the library construction protocol. These data are generally compiled by different people who typically differ in their knowledge of the requirements of the bioinformatics analysis tools, or who may not even know which bioinformatics tools will be used and lack complete information about the end-to-end study design. For example, a study coordinator may compile per-subject demographic information, a clinician may compile medical information, a lab technician may compile information about the DNA extraction and sequencing and a bioinformatician may compile any missing minimum-standards-compliance information. As a result, the most time-consuming step in a bioinformatics analysis is often merging these data from different human-generated sources and bringing them into compliance with the format specifications of the bioinformatics program(s) that will be used.

Users generally manage their tabular data in spreadsheet programs (e.g. Microsoft Excel). This is convenient for researchers who are compiling the requisite information (the study coordinators, clinicians, etc.) because the spreadsheet programs can easily be used on different platforms including laptops and tablets, and because they provide a familiar interface. However, a common issue arises when multiple people are responsible for compiling different information for a tabular document. Versions of the document rapidly become out-of-sync, for example if a clinician and a study coordinator are adding information at the same time, or if one person accidentally adds information to an outdated version of the file. Cloud-based spreadsheet programs that allow concurrent editing, such as Google Sheets [4], can alleviate these issues because there is always one definitive version of the document that can be edited by multiple users at the same time. For this reason, among others, Google Sheets is becoming increasingly popular for creating, editing and managing human-generated tabular files used in bioinformatics analyses.

A bioinformatics package will often include a file format validator as part of its suite of tools, but validating files can be cumbersome. The user will typically export their tabular data from their spreadsheet program in the format expected by the validator (e.g. CSV or TSV), run the validator and then return to their spreadsheet program to correct errors. After correcting errors, they again export their data from the spreadsheet program, re-run the validator and repeat the process until no more errors are present. If data needs to be corrected or added to the tabular data file at some point in the future, the process will be repeated (and all old versions of that file would need to be updated). For example, this is how the sample metadata mapping file validation workflow has traditionally been performed for QIIME through versions 1.9.1 [5] (QIIME is a widely used bioinformatics package for microbiome analysis that is developed and maintained by the authors of this paper, among others). In addition to being slow, this workflow can easily result in many different versions of the sample metadata mapping file, which frequently leads to confusion about which is the latest or definitive version of the file.

### Introducing Keemei

**Fig. 1:**
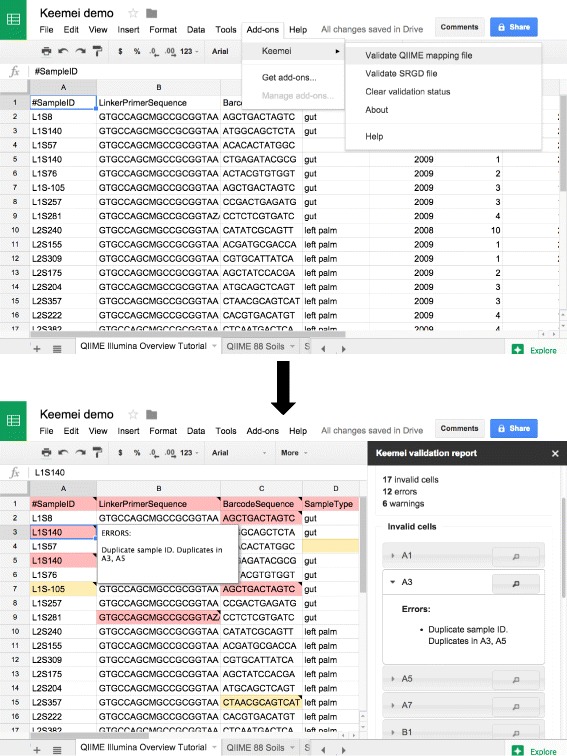
Keemei screenshots of a user validating a QIIME sample metadata mapping file. From the Google Sheets menu bar, the user selects *Add-ons > Keemei > Validate QIIME mapping file* to validate their spreadsheet against the QIIME (quantitative insights into microbial ecology) sample metadata mapping file format specification. Validation results are displayed directly in the spreadsheet and in a sidebar interface on the right side of the spreadsheet. Invalid cells are highlighted in the spreadsheet, where a red background color indicates the cell has one or more errors or warnings associated with it, and a yellow background color indicates the cell has one or more warnings associated with it. Hovering the mouse over a cell will display the reason(s) why the cell is invalid. The sidebar contains a summary of the validation (e.g. file format validated against, number of invalid cells, etc.) and lists invalid cells. The user can click on a cell in the sidebar to view the reason(s) why the cell is invalid (similar to hovering over a cell in the spreadsheet). In this screenshot, the user has clicked on invalid cell A3; we see that cells A3 and A5 contain duplicate sample identifiers, which are disallowed in QIIME mapping files. The data in this screenshot are derived from [13]

**Fig. 2:**
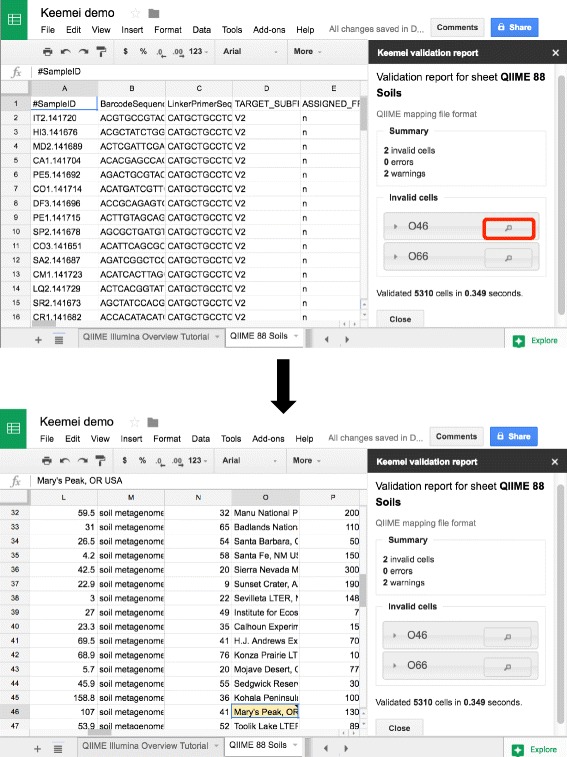
Keemei screenshots of a user focusing on an invalid cell in a QIIME mapping file. Keemei’s sidebar provides a way to focus on an invalid cell in order to correct it. This feature is especially useful when working with large sheets that would require scrolling to find and correct invalid cells. By clicking on the magnifying glass next to invalid cell O46 in the sidebar (red border added for clarity), cell O46 is made active and the user is scrolled to the cell’s location in the spreadsheet to make any necessary corrections. The data in this screenshot are derived from [14]. QIIME, quantitative insights into microbial ecology

We present Keemei, a Google Sheets Add-on for validating tabular files used in bioinformatics analyses. Keemei is available free of charge from Google’s Chrome Web Store [6]. Keemei can be installed and run on any web browser supported by Google Sheets, so browser support is not limited to Google Chrome. After installation, users are provided with a new menu option in their Google Sheets to perform tabular data validation for specific bioinformatics file formats. When a user validates their tabular data, a report in a sidebar indicates whether there are any errors or warnings in the file and, if so, which cells contain errors or warnings. Invalid cells are also highlighted directly in the spreadsheet, and hovering the mouse over a cell will display the reason(s) why the cell is invalid (Fig. 1). The user can click on a cell to view the reason(s) why the cell is invalid, and also has the option to navigate directly to a cell in the spreadsheet in order to fix it. This feature is especially useful for navigating large spreadsheets that would require scrolling to find and correct invalid cells (Fig. 2). The user can correct invalid cells and then re-validate the file, repeating the process as necessary until there are no more errors or warnings. While this process is iterative, it all occurs within the Google Sheets interface, avoiding repetitive, time-consuming and error-prone importing and exporting of files for external validation. Since there is no need to export to a new file each time the validator is run, a single definitive spreadsheet can be maintained throughout the lifetime of a project, avoiding multiple versions of exported spreadsheets.

### Benefits of a cloud-based plugin

Building Keemei as a Google Sheets Add-on provides several additional benefits over a stand-alone validator, or a plugin for a non-cloud-based spreadsheet program such as Microsoft Excel. First, as noted above, cloud-based spreadsheet programs that allow concurrent editing by multiple users assist with keeping versions of files synchronized. Therefore, building Keemei on top of a cloud-based program allows for validation of tabular file formats in the same interface that we recommend to be used for data entry and correction of errors. Next, Keemei is largely platform independent; it can be used on any system that can run Google Sheets and does not, for example, require installation of the bioinformatics software that will ultimately be used for data analysis. This cloud-based mechanism of interacting with software is increasingly popular in bioinformatics, as installation is typically trivial or not required, the burden of maintenance and upgrades is shifted from the user to the developer and in many cases it results in a graphical interface for software that previously had only a command line interface. This is exemplified in the many applications that now support Galaxy wrappers [7]. Next, Google Sheets has built-in versioning support so that it is possible to revert to previous versions of the spreadsheet. This is useful for determining if or when errors may have been introduced into tabular data. In addition, users will not need to install new versions of Keemei as it is released. When the developers push a new version to the Chrome Web Store, it is automatically updated in users’ Google Sheets environments. Finally, there are many other relevant tools being developed and released for Google Sheets, so Keemei users will have access to other useful functionality within this interface. For example, users can easily obtain graphical summaries of their data using the *Explore* function that is built into spreadsheets (e.g. a patient age histogram can automatically be generated from their sample metadata mapping files), or tag their metadata with relevant ontology terms using OntoMaton [8].

### Drawbacks of a cloud-based plugin

There are a couple of drawbacks to developing Keemei as a Google Sheets Add-on that should be noted. First, Google limits the size of spreadsheet that can be loaded (at the time of writing, around 2 million cells). Depending on the data to be validated, this may or may not be a problem. Next, being cloud-based, it is possible that Institutional Review Boards (IRBs) or other ethics or data management committees may disallow the use of Keemei for studies involving human subjects research or confidential research, even if the data is not made public. Researchers should discuss the use of Google Sheets with their IRB or other relevant bodies prior to starting their study. One step that could be taken to alleviate IRB concerns would be to ensure that no personally identifying information is contained in the data files loaded in Google Sheets.

### Current and future file format support

Keemei currently supports the validation of two specific tabular bioinformatics file formats: the QIIME sample metadata mapping file [9] and the Spatially Referenced Genetic Data (SRGD, also known as SRGD.csv) file. Both of these are used and/or generated by multiple bioinformatics programs, including QIIME, geneGIS [10] and Wildbook [11]. Keemei is designed to support the inclusion of additional format validators, and others will be added in the future, including ISA-TAB format. Future versions of Keemei will give users the ability to configure a format’s validation rules in order to modify validation stringency (e.g. disabling errors about duplicate barcode sequences if validating a QIIME mapping file constructed from several individual studies). Future versions of Keemei will also allow users to create their own ‘formats’, or sets of validation rules, from a graphical user interface, allowing validation of data formats not explicitly supported by Keemei.

It is important to note that Keemei’s validation rules are specific to each file format, and that Keemei only validates data against a file format’s specification. For example, the QIIME sample metadata mapping file format places restrictions on certain fields that are expected to contain valid DNA sequences, but does not place restrictions on other columns, such as a ‘Date_Time’ column containing invalid datetimes. In this example, Keemei would not detect invalid datetimes in the ‘Date_Time’ column if validating the sheet as a QIIME mapping file, but *would* detect invalid cells if the same sheet were validated as an SRGD file, whose file format specifies that the ‘Date_Time’ column *must* contain valid datetimes in a specific format.

### Scalability

**Fig. 3:**
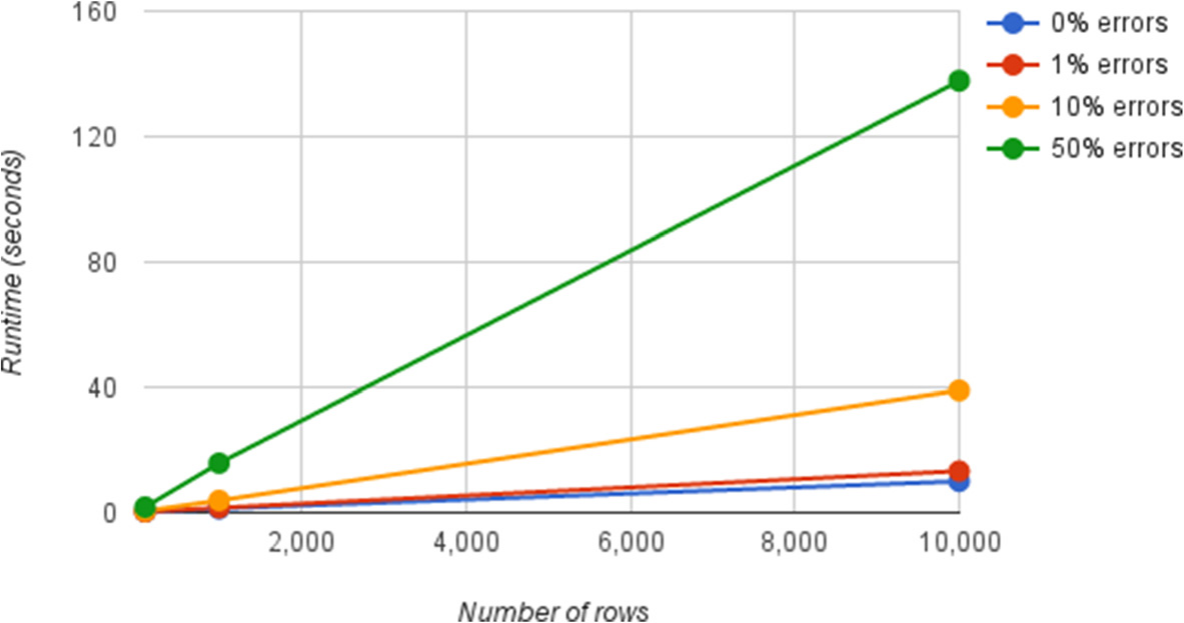
Validation performance as dataset size and error rate increases. Keemei’s validation runtime (walltime measured in seconds) is plotted against an increasing number of spreadsheet rows (dataset size) with a fixed number of columns (24). Each dataset size contains simulated QIIME (quantitative insights into microbial ecology) mapping file data with a varying percentage of invalid cells. A description of the simulated data is provided in the main text and the simulated data is available in Additional file 1

We evaluated Keemei’s performance as dataset size and error rate increase. We see a near-linear increase in validation runtime (walltime) as the number of spreadsheet rows increases for each error rate (Fig. 3). Validations were performed using simulated data in QIIME’s sample metadata mapping file format. As the number of biological samples (i.e. rows) are typically increasing in microbiome studies, simulated spreadsheets were created with a varying number of rows and exactly 24 columns representing various metadata about each sample. For each dataset size, a simulated spreadsheet was created with ‘typical’ valid data that one would see in a QIIME mapping file. These data include unique sample identifiers, unique barcode sequences of a fixed length, a commonly used 16S linker primer sequence, several columns containing unique values (e.g. simulating continuous metadata) and several columns containing a single value (i.e. columns without variance). Each *valid* simulated spreadsheet had a number of errors introduced randomly throughout the sheet to obtain the desired error rate. The type of error introduced to a cell was also random, with three possible error types: making a cell empty, introducing leading and/or trailing whitespace to a cell and introducing invalid characters to a cell. The simulated spreadsheets are available in Additional file 1.

**Fig. 4:**
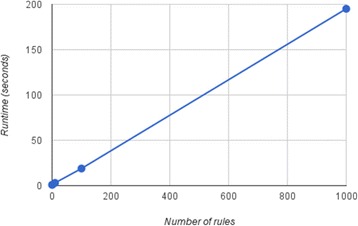
Validation performance as the number of validation rules increase. Keemei’s validation runtime (walltime measured in seconds) is plotted against an increasing number of validation rules applied to simulated data. The dataset size and error rate are held constant. A description of the simulated data is provided in the main text and the simulated data is available in Additional file 1

Keemei supports validating multiple file formats, each with their own number of rules, and we plan to allow users to define their own ‘formats’, or sets of rules, in the future. Since the number of rules affects validation performance, we evaluated runtime as the number of rules increased. A single validation rule (identifying cells with invalid characters) was applied an increasing number of times to one of the *valid* simulated spreadsheets described above (1000 rows × 24 columns, 0 % errors, part of Additional file 1). The validation rule itself was not varied so that each rule had a fixed performance cost and the performance cost as the number of rules increased could be compared. We see a near-linear increase in runtime as the number of validation rules increases (Fig. 4), indicating that Keemei should scale as more constrained formats are added in the future.

Keemei includes a ‘Developer tools’ submenu, with tools to create simulated QIIME mapping file data and execute performance benchmarks, allowing users and developers to reproduce the performance benchmarks presented here.

## Conclusions

Keemei will save researchers time and frustration by providing a convenient interface for tabular bioinformatics file format validation in a spreadsheet interface that they are already familiar with. It will allow everyone involved with data entry for a project to easily perform validation of their data, reducing the validation and formatting bottleneck that is often encountered when human-generated data files are first used with a bioinformatics system. We additionally hope that the availability of Keemei and other Google Sheets Add-ons, such as OntoMaton, will encourage a shift away from locally installed software for data management and processing toward cloud-based solutions, where multiple users can access and operate on the same files at the same time. Simplifying the tracking of essential tabular data files, such as sample metadata, will reduce common errors and thereby improve the quality and reliability of research outcomes.

### Availability and requirements

**Project name:** Keemei.

**Project home page:**http://keemei.qiime.org.

**Operating system(s):** Platform independent.

**Programming language:** Google Apps Script, JavaScript/HTML/CSS.

**Other requirements:** Web browser supported by Google Sheets.

**License:** BSD 3-Clause.

**Any restrictions to use by non-academics:** None.

## Availability of supporting data

The dataset supporting the conclusions of this article is included within the article (and Additional file 1). Snapshots of the archival code and test data as reviewed at the time of publication are also hosted in the *GigaScience* GigaDB repository [12].

## Abbreviations

IRBs, institutional review boards; QIIME, quantitative insights into microbial ecology; SRGD: spatially referenced genetic data

## Additional file

Additional file 1: An Excel spreadsheet of simulated QIIME (Quantitative Insights Into Microbial Ecology) sample metadata containing sheets of varying size and percentage of errors. Used as exemplar data in the validation and testing described in the main text. A description of the simulated data is provided in the main text. (XLSX 5518 kb)

## References

[1] Rocca-Serra P, Brandizi M, Maguire E, Sklyar N, Taylor C, Begley K (2010). ISA software suite: supporting standards-compliant experimental annotation and enabling curation at the community level. Bioinformatics.

[2] Chase JH, Bolyen E, Rideout JR, Caporaso JG (2015). cual-id: globally unique, correctable, and human-friendly sample identifiers for comparative omics studies. mSystems.

[3] Yilmaz P, Kottmann R, Field D, Knight R, Cole JR, Amaral-Zettler L (2011). Minimum information about a marker gene sequence (MIMARKS) and minimum information about any (x) sequence (MIxS) specifications. Nat Biotechnol.

[4] Google Sheets. http://www.google.com/sheets. Accessed 21 Jan 2016.

[5] Caporaso JG, Kuczynski J, Stombaugh J, Bittinger K, Bushman FD, Costello EK (2010). QIIME allows analysis of high-throughput community sequencing data. Nat Methods.

[6] Chrome Web Store. https://chrome.google.com/webstore/category/apps. Accessed 21 Jan 2016.

[7] Goecks J, Nekrutenko A, Taylor J (2010). Galaxy: a comprehensive approach for supporting accessible, reproducible, and transparent computational research in the life sciences. Genome Biol.

[8] Maguire E, González-Beltrán A, Whetzel PL, Sansone S-A, Rocca-Serra P (2013). OntoMaton: a bioportal powered ontology widget for Google Spreadsheets. Bioinformatics.

[9] QIIME File Format Descriptions. http://qiime.org/documentation/file_formats.html. Accessed 21 Jan 2016.

[10] Dick DM, Walbridge S, Wright DJ, Calambokidis J, Falcone EA, Steel D (2014). geneGIS: geoanalytical tools and arc marine customization for individual-based genetic records. Trans GIS.

[11] Wildbook Framework for Mark-Recapture Studies. http://www.wildme.org/wildbook/doku.php. Accessed 21 Jan 2016.

[12] Rideout JR, Chase JH, Boylen E, Ackermann G, Gonzalez A, Knight R, Caporaso JG. Supporting data for “Keemei: cloud-based validation of tabular bioinformatics file formats in Google Sheets”. GigaScience. 2016; http://dx.doi.org/10.5524/100204. Accessed 23 May 2016. 10.1186/s13742-016-0133-6 PMC4906574 27296526

[13] Caporaso JG, Lauber CL, Costello EK, Berg-Lyons D, Gonzalez A, Stombaugh J (2011). Moving pictures of the human microbiome. Genome Biol.

[14] Lauber CL, Hamady M, Knight R, Fierer N (2009). Pyrosequencing-based assessment of soil pH as a predictor of soil bacterial community structure at the continental scale. Appl Environ Microbiol.

